# Echocardiographic screening to determine progression of latent rheumatic heart disease in endemic areas: A systematic review and meta-analysis

**DOI:** 10.1371/journal.pone.0234196

**Published:** 2020-06-04

**Authors:** Sarah J. Gutman, Elad Shemesh, Thomas H. Marwick, Andrew J. Taylor

**Affiliations:** 1 Baker Heart and Diabetes Institute, Melbourne, Australia; 2 Department of Cardiology, The Alfred Hospital, Melbourne, Australia; 3 Monash University, Melbourne, Australia; 4 Department of Cardiovascular Medicine, Lady Davis Carmel Medical Center, Haifa, Israel; University of Western Australia, AUSTRALIA

## Abstract

**Background:**

The World Health Organisation previously recommended routine screening in school-aged children in countries with a high prevalence of rheumatic heart disease (RHD); however, it is unclear if screening-detected (latent) valve disease will inevitably evolve to a pathological lesion. Understanding the natural history of latent RHD is essential prior to recommendation of screening in endemic areas. Studies documenting the progression of latent RHD have had contrasting conclusions about the pathogenicity of latent valvular lesions. This review provides estimates of rates of progression of latent RHD.

**Methods and findings:**

In this systematic review and meta-analysis, we searched EMBASE, MEDLINE, Global Index Medicus, Africa Wide, Cochrane Database of Systematic Reviews and Global Health Database for studies published before April 30, 2019. Study data were extracted from all studies which reported follow-up data on progression of latent valve lesions. Studies with control cohorts were used to calculate comparative prevalence ratios. This study is registered with PROSPERO, number CRD42019119427. We identified 12 studies reporting follow-up data on latent RHD for 950 people in 9 countries. The estimated pooled prevalence rate for progression per year of latent RHD was 5%/year (95% CI 2–8). Eight studies reported on the progression of borderline latent RHD with an estimated pooled prevalence of 2%/year (95% CI 0–4). Three studies included control groups. There was a significant increase in the risk of progression of valvular disease in the latent group compared with controls (RR = 3.57 (95%CI = 1.65–7.70, P = 0.001). The overall risk of bias was low. Given most studies included penicillin administration we were unable to document the natural history of latent RHD. Furthermore, we were unable to perform a sensitivity analysis to determine the effect of administering penicillin prophylaxis on progression of valve disease given prescription of penicillin was not standardised.

**Conclusion:**

Latent RHD has a slow rate of progression but it is significantly higher compared to controls, with definite latent RHD having a higher rate of progression compared with borderline latent disease. There are a massive number of individuals at risk for RHD in the developing world as well as logistical challenges of screening and delivering penicillin prophylaxis. The low rate of progression from untargeted screening may be an important consideration in resource-constrained environments.

## Introduction

While virtually eradicated from developed countries, rheumatic heart disease (RHD) causes significant morbidity and mortality in low-income and middle-income countries as well as in disadvantaged indigenous populations in developed nations. [1, 2] In contrast to the relative neglect of this condition in past decades, [3] the availability of echocardiography-based screening has piqued the interest of researchers and policy makers in determining the global burden of RHD. [1] Systematic screening with echocardiography has uncovered a high prevalence of latent RHD compared with estimates based on clinically manifest disease. [4]

In response to the number of screening studies in asymptomatic individuals, the World Heart Federation published guidelines to enable rapid detection of RHD in patients without a history of ARF. [5] The three echocardiographic categories: ‘definite RHD’, ‘borderline RHD’, and ‘normal’ provide a standardised template for screening. The World Health Organization previously advocated for screening for RHD in endemic countries [6]. However, it is unclear whether screening is a worthwhile exercise outside of prevalence estimation, given the natural history of screening-detected definite and borderline RHD (together, termed latent RHD) is unknown, [7] especially in mild cases. Echocardiography-based screening is more effective than clinical screening [4] but it is also more expensive and logistically more challenging. Furthermore, the sequelae of screening are far from inconsequential with the current Australian guidelines recommending administration of intramuscular penicillin every 28 days for 5 years following diagnosis of RHD or until age 21–40 years, depending on the severity of the lesion. [8]

Studies reporting follow-up data in patients with latent RHD have had conflicting results [9] with some reporting predominantly stability and improvement [10–13] and others showing heterogenous outcomes with significant progression, development of Definite RHD, ARF and even death [14–18]. While the optimal approach of ascertaining the balance between benefits and harms of penicillin administration in latent RHD is a randomised controlled trial, there have been none so far. Given one of the fundamental requirements in evaluating a screening test is to determine whether early pathologic changes are progressive, [19] we aimed to systematically review and synthesise studies which report on the progression of latent valvular lesions. There have been two previously published meta-analyses examining the prevalence of rheumatic heart disease is endemic countries, [7] [20] including one with a section on progression of latent RHD. [20] Our search was conducted approximately 1.5 years later and includes additional cohorts. [12, 21, 22] We also present an annualised rate of progression of latent and borderline RHD and comparison with controls. The primary outcome of this study was to determine the pooled prevalence of progression of latent RHD. The secondary outcomes were to pool data on subsequent diagnosis of ARF, valve disease requiring intervention, heart failure diagnoses, all-cause mortality and adherence to penicillin if prescribed.

## Methods

### Search strategy and selection criteria

A systematic review and meta-analysis were performed. We searched Medline, Embase, Global Index Medicus (which includes Latin America and the Caribbean database LILACS as well as World Health Organisation Library Information System WHOLIS), Global Health Database, African Journals Online, and the Cochrane Database of Systematic Reviews on 30 April 2019, for screening studies on RHD with neither language nor date restriction. A sample search strategy is shown in S1 Appendix. Additionally, a manual search of all eligible articles' reference lists, articles citing eligible articles as well as relevant review articles was carried out in order to identify any additional literature.

We included all primary observational studies assessing the outcomes of latent RHD in humans in countries classified as having a high/endemic incidence of RHD or specific populations with a high incidence of RHD in Western nations. Eligible studies had a cohort or case-control design. We included studies in which outcomes have been reported for screened-negative and screened-positive cases and also those which report only the results of screened-positive patients.

Once the searches were completed, the software programmes Endnote (Clarivate Analytics, Philadelphia, United States) and Covidence (Covidence, Melbourne, Australia) were used to conduct the de-duplication of citations and for the screening process. Two authors (SJG and ES) screened all titles and abstracts, reviewed full-text articles, and assessed their eligibility for inclusion. Disagreements were resolved by discussion and a final decision was reached after agreement between the reviewers.

### Data extraction

All data were independently extracted by two reviewers (SJG and ES). Discrepancies were resolved by mutual consensus. We extracted basic demographic data (country of study, age and sex), echocardiographic criteria used, prescription of and adherence to secondary penicillin prophylaxis as well as the prevalence of latent RHD. For studies using the 2012 WHF echocardiographic criteria, prevalence of latent RHD was further divided into prevalence of definite and borderline disease. We extracted length of follow up for each study and summary estimates for percent of valve lesions which remained stable, regressed and progressed. Studies with control groups using 2012 WHF criteria were used to calculate a pooled risk ratio of progression of latent RHD in screening positive cases compared with controls. The study quality was assessed as part of the data extraction strategy by two reviewers (SJG and ES) with the Standardised Risk of Bias Tool [23] designed to assess bias in population-based prevalence studies. Items were rated as either low risk or high risk. All items rated as low risk were added to calculate an overall score whereby a higher score indicated stronger methodology and a lower risk of bias.

### Statistical analysis

Given we were not evaluating the effect of an intervention, we dealt with progression as a proportion and therefore it was appropriate to perform a meta-analysis of prevalence. We combined the extracted prevalence and risk ratio data in a random-effects meta-analysis in Stata version 15.1 (StataCorp, College Station, United States) with the metan [24] command. We estimated heterogeneity using the I^2^ statistic which we interpreted as low (25%), moderate (50%) or high (75%). [25]

We performed sub-group analysis on pre and post 2012 WHF criteria. We performed a sensitivity analysis on progression of valvular disease in borderline cases by removing 4 studies which did not report on the progression of borderline RHD. Three studies were not included in the analysis of the primary endpoint because they did not report on the progression of definite cases or only followed up borderline cases [11], [14], [12] All three were included in the sensitivity analysis for progression of borderline cases.

### Role of the funding source

The funder of the study had no role in study design, data collection, data analysis, data interpretation, or writing of the report. SJG, AJT, THM and ES had full access to all the data in the study and had final responsibility for the decision to submit for publication.

### Deviation from the protocol

As most of our studies did not have a control group we used the standardised Risk of Bias Tool which specifically assesses the risk of bias in prevalence studies. [23] Given the small number of comparative studies uncovered in our search we were unable to generate a meaningful funnel plot to assess for publication bias. [26] The planned subgroup analyses on patients by age, type of echocardiographic abnormalities and adherence to penicillin if prescribed was not performed due to lack of information in the studies. [27]

## Results

**Fig 1:**
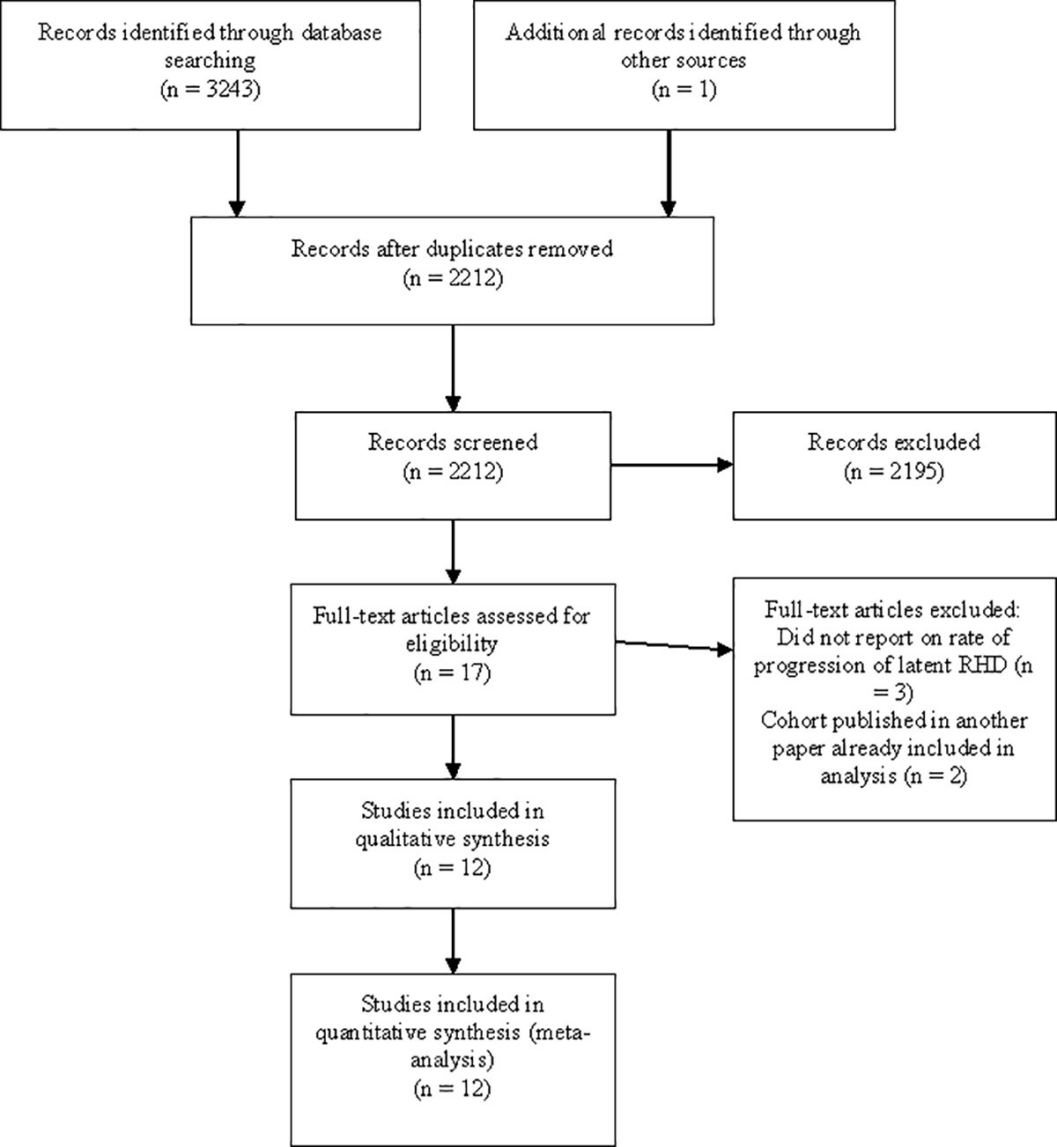
PRISMA [28] flow diagram for systematic review.

We identified 3244 publications, 17 of which were potentially eligible (Fig 1). Twelve articles describing 12 populations in 9 countries met the inclusion criteria and were included in the systematic review and meta-analysis. Among the populations, 5 were from Africa, 4 from Oceania, two from Asia and one from Latin America. The median age of the study population at follow up, as reported in 7 studies, was 12.2 years (IQR 10.8–18), and the median percentage of female subjects, as reported in 10 studies, was 59.3% (IQR 55–66) (Table 1).

**Table 1:** Screening trials meeting inclusion criteria.

Author, Year	Country	Number screened	Number (%) borderline	Number (%) Definite	Number (%) latent (pre 2012 WHF)	Number (%) latent followed up	Female (%) latent	Age at follow up, years(mean/median)	Length of follow up, months(mean/median)	Echo criteria used	Definition of progression	Blinded to original study	Secondary prophylaxis prescribed	Group prescribed prophylaxis	Secondary prophylaxis adherence
Paar(2010) [21]	Nicaragua	3150	NA	5(0.2)^	150(4.8)	134(89)	83(55)	10.7 (median)	5.7 (median)	NIH-WHO	Worsening of NIH-WHO diagnostic category	NR	NR	NR	NR
Bhaya(2011) [13]	India	1059	NA	NA	54(5.1)	54(100)	NR	NR	24 (NR)	Consensus among experts	Significant regurgitation of mitral and/or aortic valves [29]	NR	100%	NR	NR
Saxena(2011) [10]	India	6270	NA	NA	128(2)	100(78.1)	NR	10.8 (median)	15.4 (mean)	Modified WHO	Worsening in grade of valvular regurgitation	NR	Yes	Only if moderate MR	NR
Beaton(2014) [17]	Uganda	4869	61(1.3)	11^#^	N/A	51(71)	32(63)	9.6 (median) §	25 (median)	WHF	Worsening of WHF diagnostic category	Yes	23%	Definite RHD, probable RHD, advancing lesion	85.5%
Rémond(2015) [14]	Australia	Subset of children from an earlier study [30] [31]	55(1.4)	NR	N/A	55(100)	69(59.0)	13.7 (median)	44 (median)	WHF	New morphological/functional abnormality or progression of severity of a functional valve lesion	Yes	18.8%	NR	NR
Mirabel(2015) [32]	New Caledonia	17633	49(0.28)*	41(0.23) *^#^	114(0.65)	114(100)	60(52.6)	12.2(median)	31 (median)	New Caledonia Criteria	Increased grade of valve disease and/or newly diagnosed MR, AR and/or MS	Yes	NR	NR	88.6%
Zühlke(2016) [16]	South Africa	2720 (from previous publication) [33]	42(1.5)	13^#^	N/A	44(80)	29(65.9)	18(median)	60.2 (median)	WHF	Worsening of WHF diagnostic category	NR	NR	NR	4.6%
Engelman(2016) [15]	Fiji	NR	17	20^#^	N/A	37	66(67.4)	17(median)	90 (mean)	WHF	Worsening of WHF diagnostic category, increase in severity of definite cases, valve surgery	Yes	59.2%	Definite RHD	low adherence across the cohort‡
Bertaina(2017) [11]	New Caledonia	8684	25(0.3)	NR	N/A	25(100)	17(68)	9.8y (mean) §	23 (median)	WHF	Progression to definite RHD and/or increased grade of valve disease	Yes	NR	Borderline	24%
Beaton(2017) [18]	Uganda	1715	164(9.6)	63^#^	N/A	227(100)	137 (60.4)	12(median)	28.8 (median)	WHF	Worsening of WHF diagnostic category, worsening in the severity of regurgitation at the mitral or aortic valve, development of new MS or an increase in grade of MS, death attributable to RHD	Yes	49.3%	Borderline and definite	84.7%
Kotit(2017) [22]	Egypt	3062	35(1.1)	60(1.2) ^#^	N/A	72(75.8)	34(47.2)	14.1 (mean)	42.1 (mean)	WHF	Development of structural/functional abnormality or progression of severity of functional valve lesion	Yes	Yes	Borderline and definite	83.3%
Sanyahumbi(2019) [12]	Malawi	1450	37(2.6)	11^#^	N/A	48(98)	6(55) †	9.97(median) §	23.5 (mean)	WHF	Worsening of WHF diagnostic category	NR	Yes	Definite RHD	18%

NIH = National Institute of Health; WHO = World Health Organisation; WHF = World Heart Federation; NA = not applicable as study published pre-publication of 2012 WHF criteria; NR = not reported; MR = mitral regurgitation; AR = aortic regurgitation; MS = mitral stenosis.

^ = Defined according to NIH-WHO criteria

# = Defined according to WHF criteria

* The initial cohort was reclassified according to WHF criteria

† = Number of females only recorded for definite RHD cases, not for borderline cases

‡ = only 2(2%) had ≥ 80% adherence

§ = Age at screening (age at follow up was not reported).

**Fig 2:**
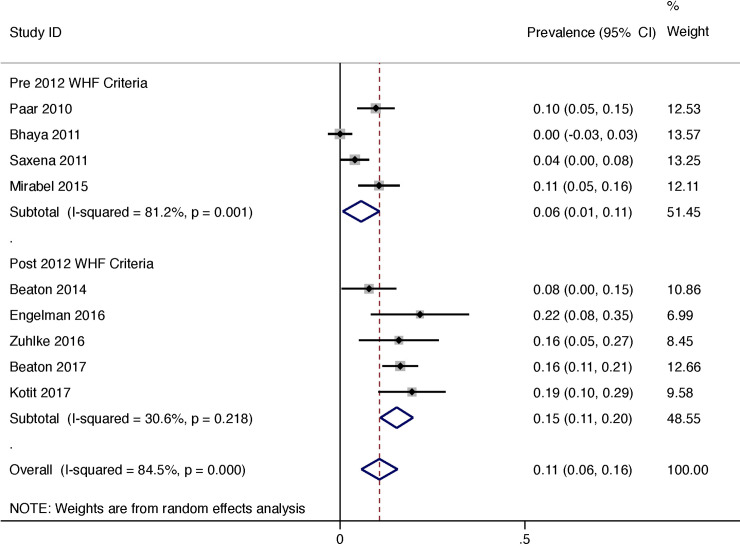
Progression over duration of study of latent rheumatic heart disease divided by sub-group (pre-publication of 2012 WHF criteria and post publication of 2012 WHF criteria).

The progression of latent RHD was reported in 9 studies and was 11% (95% CI 6–16) in total, 6% (95% CI 1–11%) using non-WHF criteria and 15% (11–20) using the 2012 WHF criteria (Fig 2) for the entire cohort over the duration of the study (follow up range 5.7–90 months). The heterogeneity of reported prevalence was high between all studies (I^2^ = 84.5%, P<0.001).

**Fig 3:**
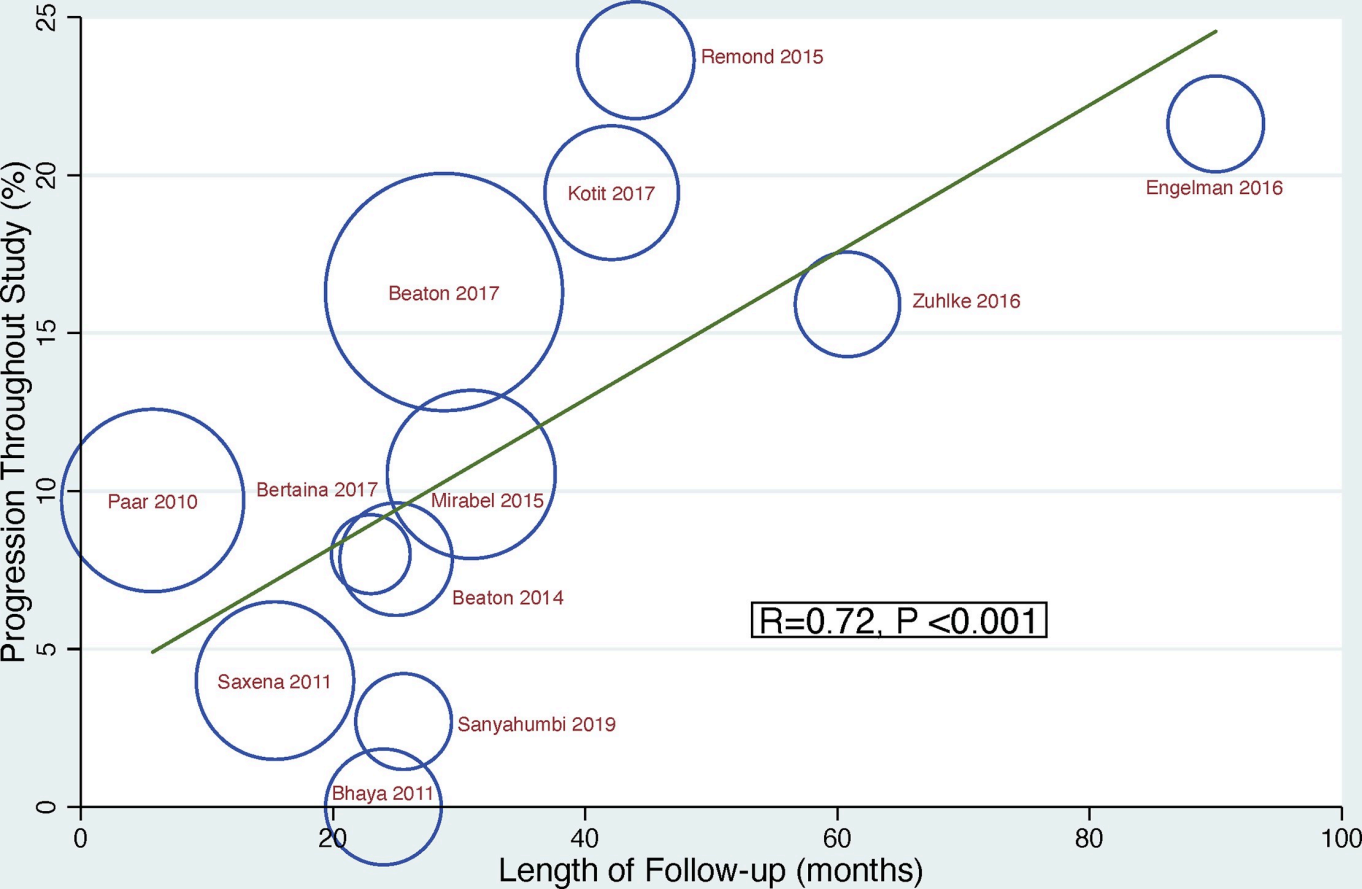
Correlation between length of study and progression of latent RHD weighted by sample size.

**Fig 4:**
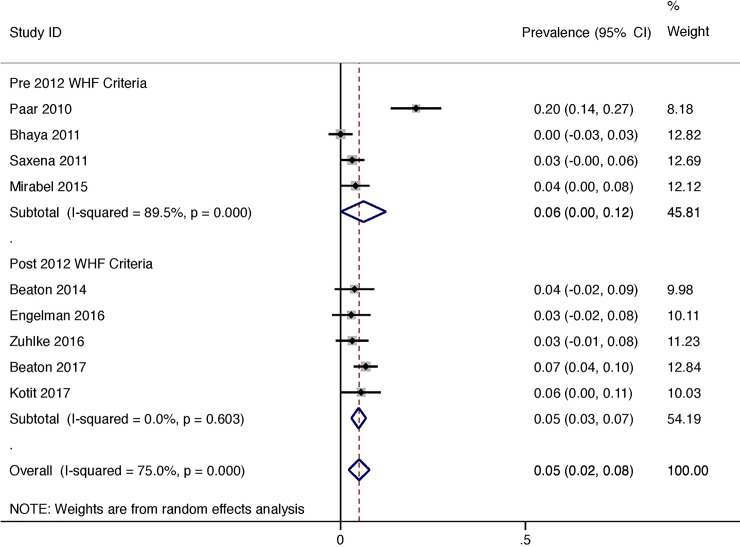
Latent rheumatic heart disease: Progression per year.

Given there was a linear correlation between length of follow-up and progression of latent RHD throughout duration of study (R(s) = 0.72, P = <0.001, weighted for sample size, Fig 3) we estimated an annualised progression rate for latent RHD. The progression of latent RHD per year was 5% (95% CI 2–8) per year in total, 6% (95% CI 0–12) using the non-WHF criteria and 5% (95% CI 3–7) using the 2012 WHF criteria (Fig 4). The heterogeneity of reported prevalence was high between studies (I^2^ = 75.0%, P = <0.001).

**Fig 5:**
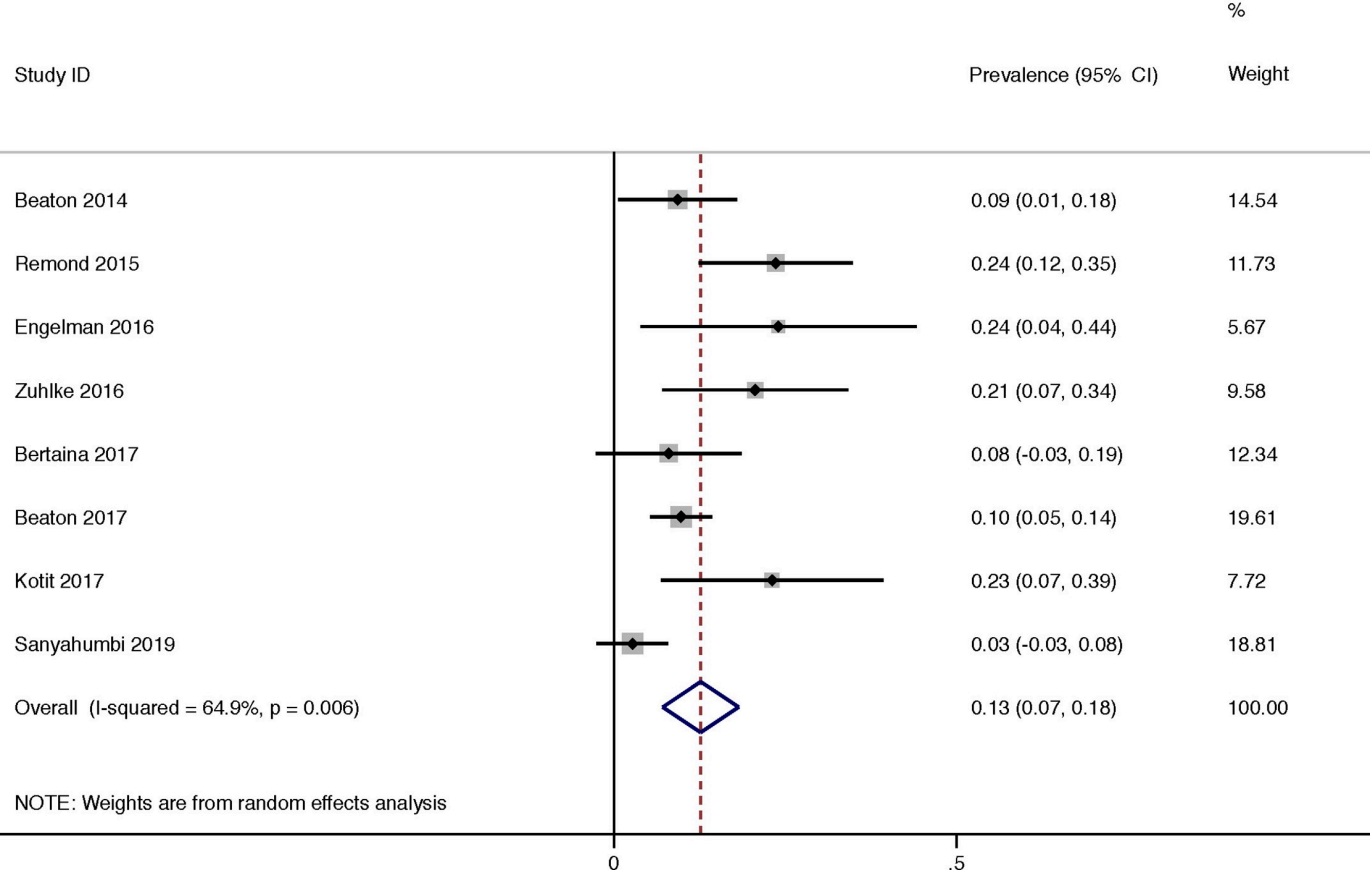
Progression over duration of study of borderline rheumatic heart disease.

**Fig 6:**
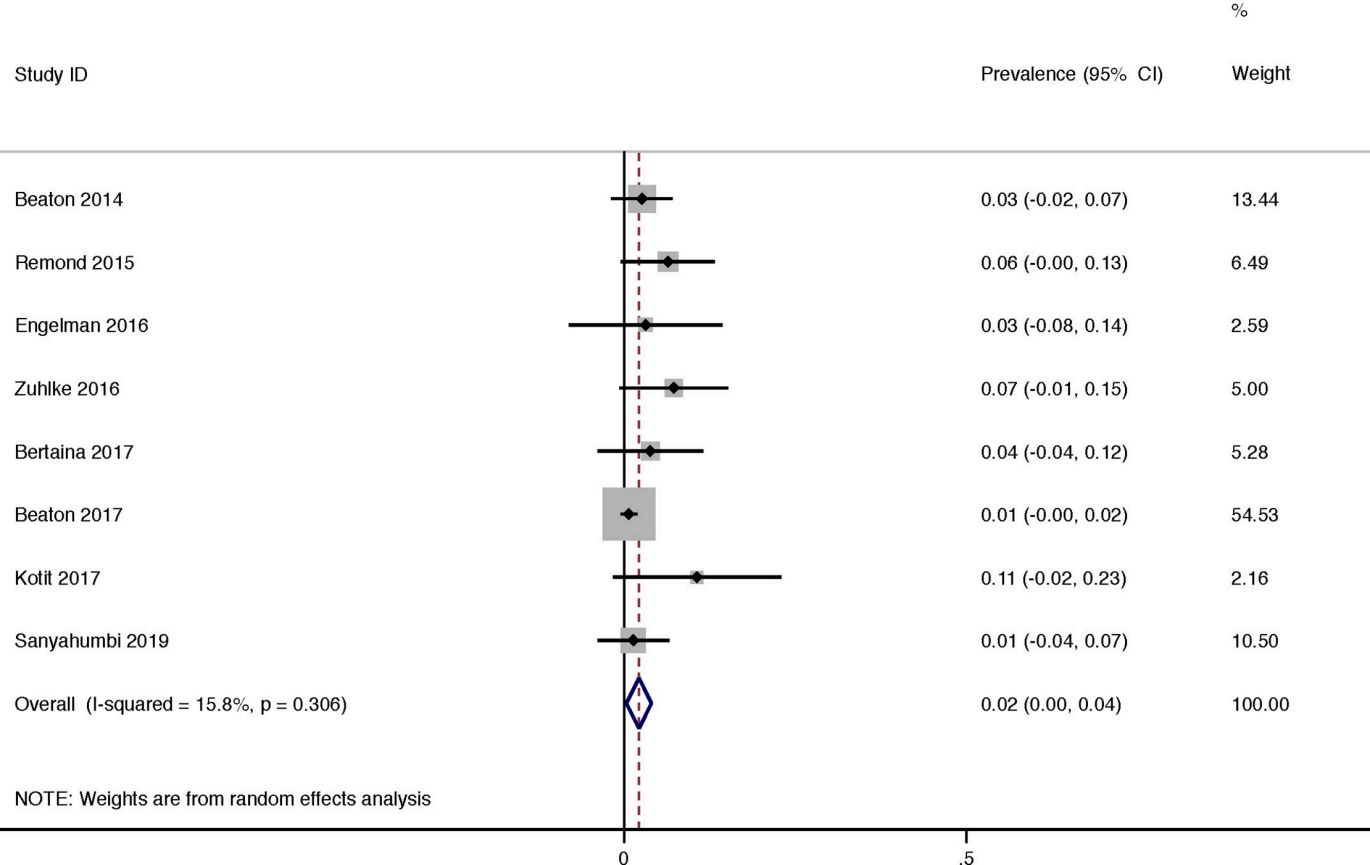
Borderline rheumatic heart disease: Progression per year.

The progression of borderline RHD was reported in 8 studies and was 13% (95% CI 7–18) with a moderate heterogeneity of reported progression between all (I^2^ = 64.9%, P = 0.006) (Fig 5). The progression of borderline RHD per year was 2% (95% CI 0–4) with a low heterogeneity of reported progression between all (I^2^ = 15.8%, P = 0.31) (Fig 6).

### Risk ratio of progression of RHD

**Fig 7:**
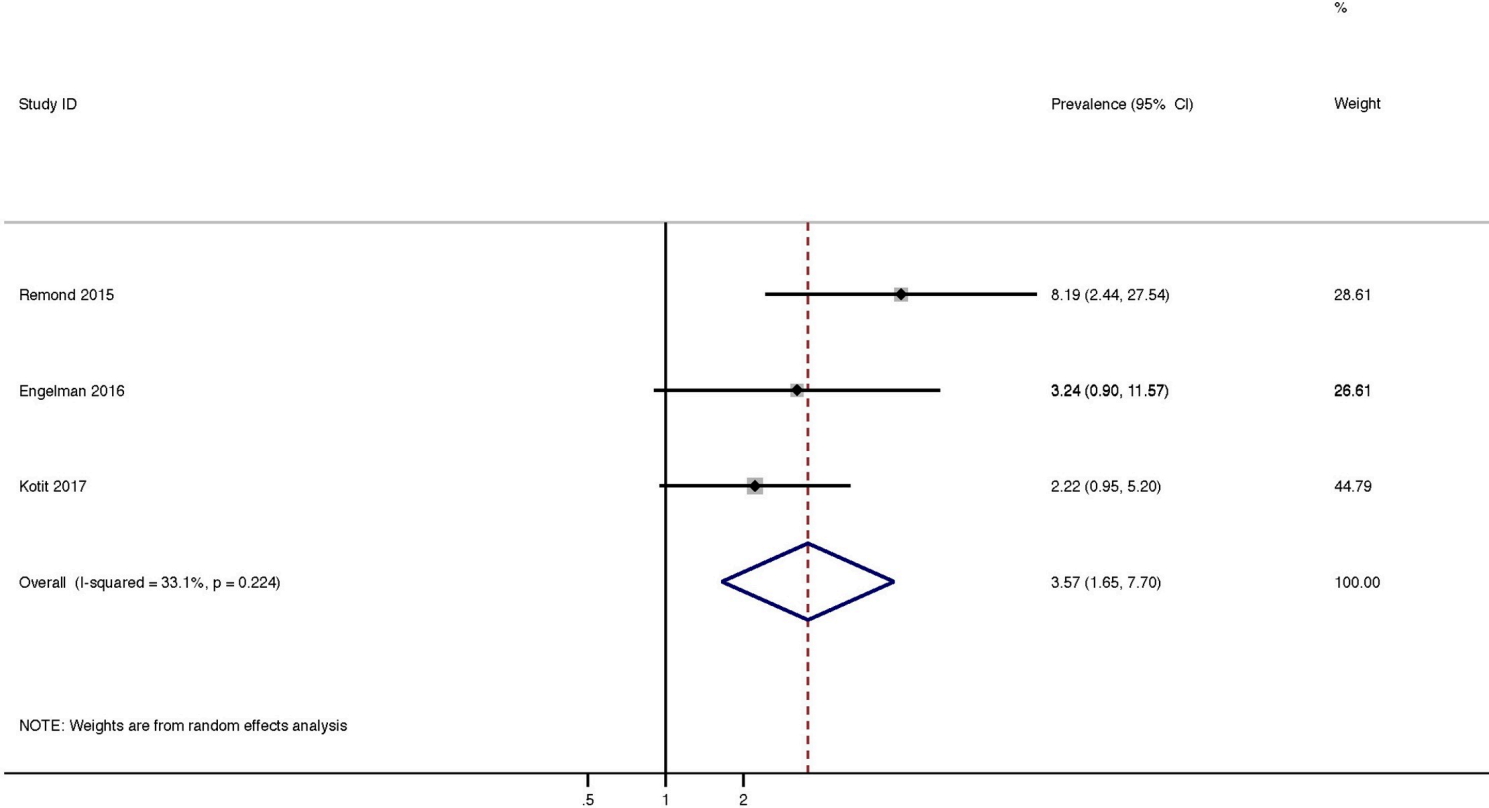
Pooled risk ratio of progression of latent RHD vs controls.

Three studies using the 2012 WHF echocardiographic criteria included control groups which allowed us to calculate a pooled risk ratio for progression of latent RHD compared to controls. The risk ratio for progression of valvular lesions in those with latent RHD compared to controls was 3.57 (95%CI = 1.65–7.70, P = 0.001) (Fig 7).

**Fig 8:**
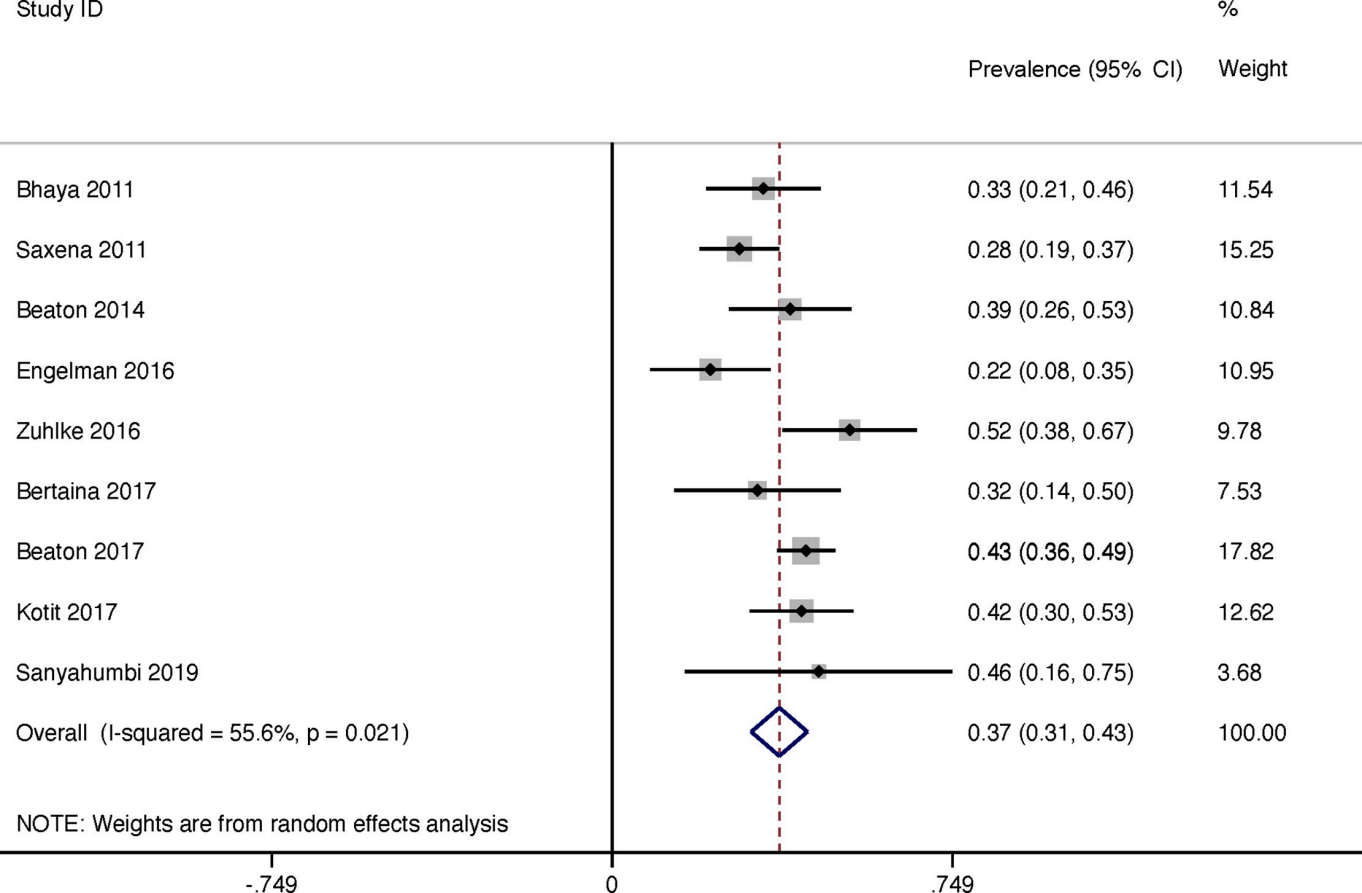
Regression over duration of study of latent rheumatic heart disease.

**Fig 9:**
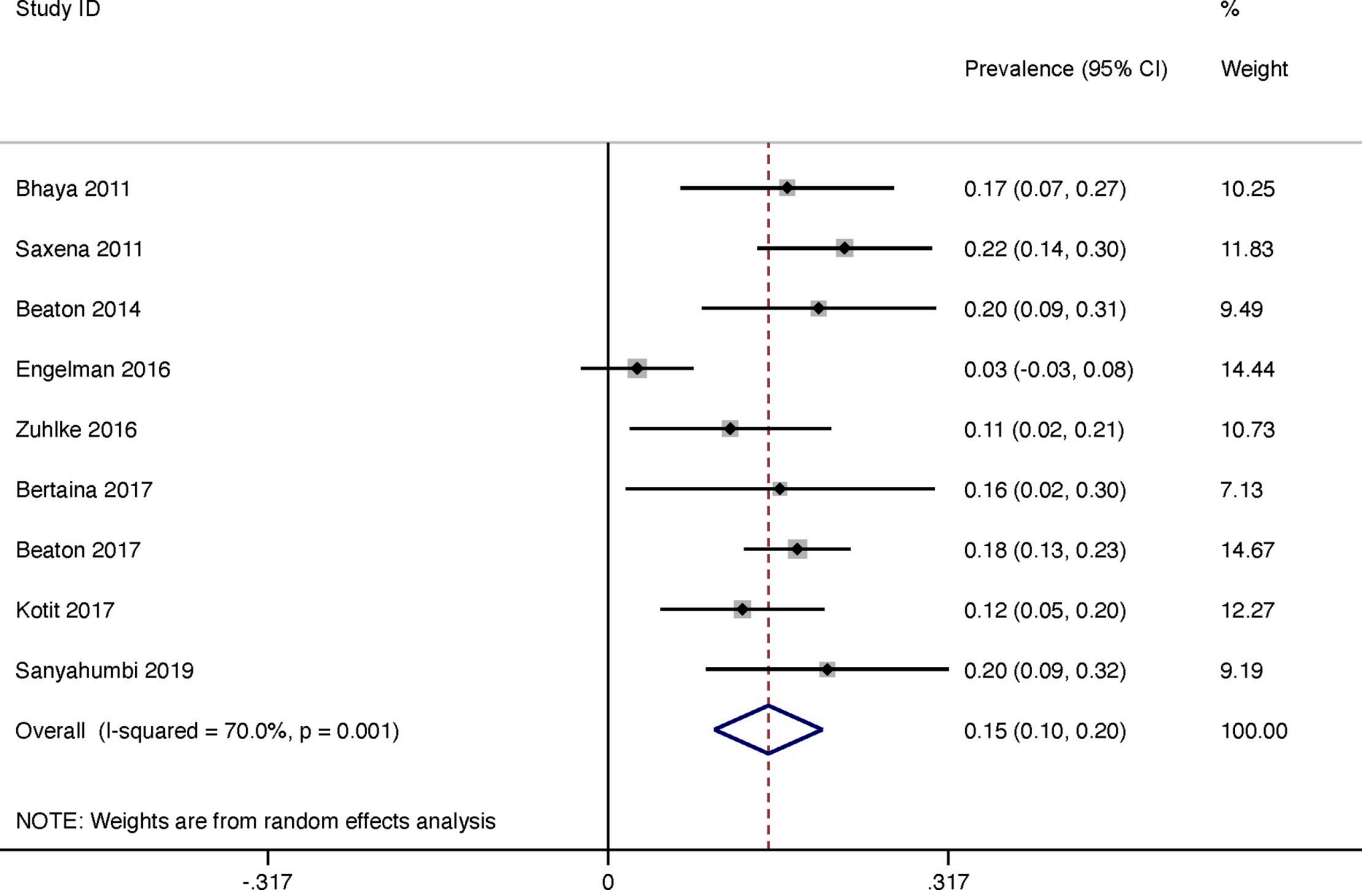
Latent rheumatic heart disease: Progression per year.

The rate of regression of latent RHD was reported in 9 studies and was 37% (95% CI 31–43) in total for the duration of the study (Fig 8). The heterogeneity of reported prevalence was moderate between all studies (I^2^ = 50.7%, P = 0.032). There was a linear correlation between length of follow-up and regression of latent RHD throughout duration of study (R(s) = 0.45, P = <0.001, weighted for sample size). We estimated the annualised regression rate for latent RHD per year to be 15% (95% CI 10–20) with a moderate heterogeneity between studies (I^2^ = 70.0%, P = 0.001) (Fig 9).

### Secondary outcomes

We were not able to perform meta-analysis of secondary outcomes due to lack of data and therefore present a narrative review of pre-specified secondary outcomes.

#### Subsequent diagnosis of ARF

In Beaton et al’s 2014 study 2 children developed ARF during the study period. One, a 10-year-old girl was in the borderline group and was not receiving penicillin prophylaxis. The second, an 11-year-old boy, had received a diagnosis of definite RHD and was prescribed monthly penicillin to which he described 100% adherence. Mirabel et al reported an incidence of ARF of 10.28/1000/year. Remond et al reported 9 episodes of ARF during the follow up period. Beaton et al’s 2017 study and Saxena et al’s study both reported no cases of ARF during the follow up period.

#### Valve disease requiring intervention

Four studies reported on this outcome. Zuhlke et al described a 22-year-old girl who was HIV positive with borderline RHD at screening who had severe MR at follow up with LV dilatation and reduced ejection fraction. She not been prescribed penicillin. Engelman et al reported that 4 patients in their cohort required valvular surgery. Beaton et al’s 2017 study and Mirabel et al’s study reported that there were no cases requiring valvular intervention in the follow up period.

#### Heart failure diagnoses

Three studies reported on this outcome. Zuhlke et al described a 16-year-old girl with definite RHD who presented in heart failure at 8 weeks of pregnancy. She had been adherent to monthly benzylpenicillin. The second child with a diagnosis of ARF described in Beaton et al’s 2014 study was hospitalised due to severe congestive cardiac failure in the setting of ARF. Mirabel et al reported one episode of heart failure during the follow up period.

#### All-cause mortality

There were 2 studies which reported on death in the follow up period. Beaton et al’s 2017 study reported that 2 patients (9.5% of patients diagnosed with moderate/severe definite RHD) died. Mirabel et al reported no deaths. Engelman et al reported that 2 RHD cases from their screening cohort (1.5%) died from severe RHD prior to receiving a follow up echocardiogram.

#### Factors associated with progression

In this review heterogeneity was too high for a meta-regression. Seven studies reported on factors associated with persistence or progression of latent RHD. Beaton et al in their 2014 and 2017 studies study found that younger children were at a higher risk of an unfavourable outcome whereas in Kotit et al’s study, it was reported that older children exhibited higher rates of progression. Other risk factors for progression were: a greater number of mitral valve morphologic changes, [17] [18] [22] a higher anti-streptolysin O titre, [17] overcrowded living conditions, [32] pathological mitral regurgitation, [16] more advanced disease category, [18] and female sex. [22] Engelman et al found that only longer follow up (>5 years) was associated with improvement in echocardiographic diagnosis and Bertaina et al, who followed up borderline cases only, did not find any risk factors for progression.

### Risk of bias

A summary of the risk of bias of the included articles is provided in Table 2. Ten studies (83%) were considered to be at low risk of bias, and 2 studies were considered to be moderate risk (17%), one due to lack of standardised echocardiographic criteria [13] and one due to short follow up time. [21] Furthermore, there were four studies [21] [13] [12] [10] which did not report whether echocardiography reporters were blinded to the diagnosis in the original studies which has the potential to introduce significant bias.

**Table 2:** Standardised risk of bias tool.

	Paar, 2010	Bhaya, 2011	Saxena, 2011	Beaton, 2014	Rémond, 2015	Mirabel, 2015	Zühlke, 2016	Engelman, 2016	Bertaina, 2017	Beaton, 2017	Kotit, 2017	Sanyahumbi, 2019
**Study Design**	Prevalent cohort	Prevalent cohort	Prevalent cohort	Prevalent cohort	Prevalent cohort	Prevalent cohort	Prevalent cohort	Prevalent cohort	Prevalent cohort	Prevalent cohort	Prevalent cohort	Prevalent cohort
**External validity**												
Was the study’s target population a close representation of the national population in relation to relevant variable?	Yes	Yes	Yes	Yes	Yes	Yes	Yes	Yes	Yes	Yes	Yes	Yes
Was the sampling frame a true or close representation of the target population?	Yes	Yes	Yes	Yes	Yes	Yes	Yes	Yes	Yes	Yes	Yes	Yes
Was some form of random selection used to select the sample, OR, was a census undertaken?	Yes	Yes	Yes	Yes	Yes	Yes	Yes	Yes	Yes	Yes	Yes	Yes
Was the likelihood of non-response bias minimal?	Yes	Yes	Yes	No	Yes	Yes	Yes	Yes	Yes	No	Yes	Yes
**Internal validity**												
Were data collected directly from the subjects (as opposed to a proxy)?	Yes	Yes	Yes	Yes	Yes	Yes	Yes	Yes	Yes	Yes	Yes	Yes
Was an acceptable case definition used in the study?	Yes	No	Yes	Yes	Yes	Yes	Yes	Yes	Yes	Yes	Yes	Yes
Was the study instrument that measured the parameter of interest shown to have reliability and validity?	Yes	Yes	Yes	Yes	Yes	Yes	Yes	Yes	Yes	Yes	Yes	Yes
Was the same mode of data collection used for all subjects?	Yes	Yes	Yes	Yes	Yes	Yes	Yes	Yes	Yes	Yes	Yes	Yes
Was the length of the shortest prevalence period for the parameter of interest appropriate?	No	Yes	Yes	Yes	Yes	Yes	Yes	Yes	Yes	Yes	Yes	Yes
Were the numerator (s) and denominator r(s) for the parameter of interest appropriate?	Yes	Yes	Yes	Yes	Yes	Yes	Yes	Yes	Yes	Yes	Yes	Yes
Score	9	9	10	9	10	10	10	10	10	10	10	10
Summary item on the overall risk of study bias	Moderate	Moderate	Low	Low	Low	Low	Low	Low	Low	Low	Low	Low

## Discussion and conclusion

This is the most contemporary systematic review and meta-analysis pooling all studies reporting on progression of latent RHD and the first study to calculate an annual rate of progression for both latent definite and borderline RHD. We have also shown that latent RHD has a higher rate of progression compared to controls although the rate of regression is also very high.

In order to justify a screening program it must provide substantial benefit to the target population and must identify a significant proportion of the people at risk of developing the adverse outcome. [34] RHD is endemic throughout much of the developing world and although many attempts have been made to simplify echocardiographic protocols, screening all at risk individuals with echocardiography is a huge undertaking.

Although echocardiographic screening poses no physical risk for those with a false positive result, it is not a harmless process. Echocardiographic screening may have detrimental effects, specifically increased anxiety as well as a decrease in physical activity among those who receive an abnormal screening result. [35] Furthermore if the goal of screening is to institute secondary penicillin prophylaxis, we must determine whether prophylactic penicillin can mitigate progression in latent disease as it does in RHD diagnosed after clinically manifest ARF. [36–40] Administration of intra-muscular penicillin to children must be rigorously justified given it is painful [41] and causes significant trauma for the patient, parents and health care worker [42] with reports of significant peri-procedural anxiety, needle phobia, and medical fear. [43] Negative experiences, when experienced by large numbers of children and adolescents, amount to large harms for a population. [34]

That is not to say that there are no positives of echocardiographic screening. Given the devastating complications and high mortality rate of clinically diagnosed RHD, [44] screening may identify patients who are likely to benefit from cardiac surgery prior to the development of irreversible heart failure, infective endocarditis or stroke. [45]

While surgical intervention or prophylactic penicillin may not always be necessary, close follow up may be warranted, especially in patients in groups shown to have a greater propensity for progression. Although we were unable to perform a meta-regression, predictors for progression were extremely heterogeneous and some studies did not find any significant predictors. [11, 15] We reported that the rates of progression in latent RHD are higher than the rates of borderline disease, highlighting the greater pathogenicity of screen detected definite RHD compared with borderline disease.

Given the high rates of regression of latent RHD it is possible that some normal cases are being included in these studies and this may contribute to the very low rate of progression and lack of identifiable risk factors for progression in these patients. [46] A test to differentiate changes that are benign from changes that represent early RHD pathology would be highly desirable in this population. Ideally, a secondary test can be applied to screened-positive patients that is sensitive and more specific than echocardiography. The alternative might be to identify the subset of cases with latent disease at highest risk of progression, such as those with definite RHD, or at least to identify those at a low enough risk of progression to safely withhold penicillin prophylaxis.

Echocardiographic screening for RHD does satisfy some of the basic fundamentals of a screening test given there is a significant burden of RHD with an initial latent stage which can be detected by echocardiography which is a simple, accessible, and sensitive test. What remains to be seen is if the early stages are treatable by penicillin, and if early intervention improves prognosis in a cost-effective manner. [47] The “GOAL (GwokO Adunu pa Lutino) Trial: Determining the Impact of Penicillin on Latent Rheumatic Heart Disease” will randomise children aged 5–17 with latent RHD and compare progression of valvular lesions in children receiving penicillin prophylaxis and children not receiving penicillin prophylaxis. [48] This essential trial will hopefully address the clinical equipoise that has developed regarding penicillin administration in latent RHD.

The progression rates of latent disease shown in our study are extremely low especially when compared to the rates of lesion regression. Furthermore, in most of the included studies, even in lesions that progress, there were extremely low rates of heart failure, severe valvular disease and death Latent definite RHD was shown to have a high complication rate (20% over a median follow-up of 7 years) in another cohort but this was postulated to be a country specific finding and may have represented a high rate of “missed clinical disease”. [45] In low risk groups, the value of screening may be small, therefore like all tests, echocardiographic screening for the purpose of prophylactic penicillin administration needs to be evaluated in the context of pre-test probability. A randomised trial is the ideal way to solve this conundrum and must demonstrate feasibility, cost effectiveness and an incremental value of screening prior to its widespread implementation. [48]

### Limitations

Four studies did not utilise the 2012 World Heart Federation guidelines (Table 1). The guidelines used by these studies are less specific compared with the 2012 WHF guidelines. [49] In order to overcome this, we split the pooled analysis for the primary endpoint into pre and post 2012 WHF criteria. The studies utilising the 2012 WHF guidelines had a higher pooled rate of progression (15%) compared with the studies using the less specific criteria (6%). We believe this is likely due to the inclusion of more normal cases in the older studies. There were 2 studies which reported different grades of definite RHD. [15, 18] The results for the pooled risk ratio of progression of latent RHD vs controls (Fig 7) should also be interpreted with caution given the small number of studies with available control groups.

We were unable to analyse the different grades of latent definite RHD individually however we believe that this is an important distinction which warrants further study. The prescription of penicillin was not uniform in the studies (Table 1) and therefore we were unable to perform a sensitivity analysis to determine the effect of administering penicillin prophylaxis on progression of valve disease. There was significant heterogeneity detected in many of our analyses. We believe the most important reasons for this are variation in diagnostic criteria used across studies, different follow up periods in each study and different practices in all the countries involved. Finally, although the risk of bias in included studies was mostly low, all were prevalent cohort studies and therefore prone to certain inherent bias. [50]

Echocardiographic screening identifies a group of patients who are at a higher risk of progressive disease compared to the general community but it is not clear if the risk is high enough to justify penicillin prophylaxis. Future research will focus of better ways of further stratifying this increased-risk cohort.

## Supporting information

S1 Checklist PRISMA checklist [28]. (DOC)

S1 Appendix Search strategy for OVID. (DOCX)
