# Supplementary material for: Echocardiographic screening to determine progression of latent rheumatic heart disease in endemic areas: A systematic review and meta-analysis
Source: PLoS One. 2020 Jun 4;15(6):e0234196. doi: 10.1371/journal.pone.0234196 (PMC7272083; doi:10.1371/journal.pone.0234196)
Supplement: S1 Appendix — (DOCX) [file pone.0234196.s002.docx]

**S1 Appendix: Search strategy for OVID**

| 1. Rheumatic Heart Disease/ |
| --- |
| 2. Rheumatic Heart Disease.mp. |
| 3. 1 or 2 |
| 4. prog*.mp. |
| 5. subclinical.mp. |
| 6. sub-clinical.mp. |
| 7. latent.mp. |
| 8. undiagnosed.mp. |
| 9. missed.mp. |
| 10. borderline.mp. |
| 11. asymptomatic.mp. |
| 12. progress*.mp. |
| 13. prevalence.mp. [mp=title, abstract, heading word, drug trade name, original title, device manufacturer, drug manufacturer, device trade name, keyword, floating subheading word, candidate term word] |
| 14. (screen* adj2 detect*).mp. |
| 15. 4 or 5 or 6 or 7 or 8 or 9 or 10 or 11 or 12 or 13 or 14 |
| 16. 3 and 15 |
